# Mechanisms and insights into drug resistance in cancer

**DOI:** 10.3389/fphar.2013.00028

**Published:** 2013-03-14

**Authors:** Hiba Zahreddine, Katherine L. B. Borden

**Affiliations:** Department of Pathology and Cell Biology, Institute of Research in Immunology and Cancer, Université de MontréalMontreal, QC, Canada

**Keywords:** origin of cancer, multidrug resistance, drug metabolism, drug transporters, oncogene addiction, microenvironment, collateral sensitivity, synthetic lethality

## Abstract

Cancer drug resistance continues to be a major impediment in medical oncology. Clinically, resistance can arise prior to or as a result of cancer therapy. In this review, we discuss different mechanisms adapted by cancerous cells to resist treatment, including alteration in drug transport and metabolism, mutation and amplification of drug targets, as well as genetic rewiring which can lead to impaired apoptosis. Tumor heterogeneity may also contribute to resistance, where small subpopulations of cells may acquire or stochastically already possess some of the features enabling them to emerge under selective drug pressure. Making the problem even more challenging, some of these resistance pathways lead to multidrug resistance, generating an even more difficult clinical problem to overcome. We provide examples of these mechanisms and some insights into how understanding these processes can influence the next generation of cancer therapies.

## CANCER TALE: ITS TREATMENT AND RELAPSE

In 1961, Frei and Freireich initiated the high-dose four-drug combination clinical trial for the treatment of pediatric leukemia (Frei et al., 1965). Despite the threat imposed by administering four drugs at once, few weeks following treatment onset, children began to respond, “*the bone marrow biopsies came back one after another—all without leukemia cells. Red blood cells and white blood cells and platelets sprouted up in an otherwise scorched field of bone marrow. But the leukemia did not return*” (Mukherjee, 2010). Out of the 16 enrolled patients, 11 showed complete remission. This outstanding success, however, was short-lived. With the exception of a handful of children, all patients eventually relapsed, developing a more vigorous form of cancer that was no longer responsive to the treatment: leukemic cells had invaded the blood–brain barrier and colonized the brain “*the only place unreachable by chemotherapy....the children died one after the other-felled by virtue of the adaptation designed to protect them… it was a consequence of the body’s defense system subverting cancer treatment*” (Mukherjee, 2010). To date, this story still reflects the same tale of cancer treatment where its resistance and relapse remains a major challenge (Wilson et al., 2009). In this review we provide an overview of advances made in our understanding of the mechanisms that enable cancerous cells to adapt to and eventually overcome therapy, and how identifying these mechanisms can help circumvent resistance and improve treatment.

Despite its complex biological nature, many recent successes have been made in the treatment of cancer, including most strikingly chronic myeloid leukemia (CML) and acute promyelocytic leukemia (APL) which have met with great success as well as many cases of pediatric leukemias, Hodgkin’s lymphomas, and testicular cancers (Siegel et al., 2012). These success stories mainly relied on an increased understanding of the diverse molecular mechanisms governing tumor development. Owing to this, various anti-cancer therapies were designed to target disease-specific mechanisms that are absent in normal cells. Such strategies include (i) inhibition of a specific oncoprotein, such as targeting the oncogenic fusion proteins Bcr–Abl and PML–RARA with Gleevec and all *trans* retinoic acid (ATRA) with arsenic trioxide respectively or (ii) activation of a specific immune response against cancerous cells demonstrated by the use of interferon alpha alone or in combination with other anti-cancer drugs including 5-fluorouracil and cytarabine (Raderer and Scheithauer, 1995; Guilhot et al., 1997; Druker et al., 2001; Kreitman et al., 2001; Tallman et al., 2002; Goldman and Melo, 2003; O’Brien et al., 2003; Sawyers, 2004; Kreitman, 2006; Ferrantini et al., 2007; Chin and Gray, 2008; Sellers, 2011). Many of these drugs are currently being used in the clinic and have established positive impact on patient survival. However, a major impediment to their success is the development of therapeutic resistance which in some cases predates clinical intervention (Wilson et al., 2009). Based on tumor response to the initial therapy, cancer resistance can be broadly classified into two categories, primary and acquired (Meads et al., 2009; Lippert et al., 2011). While primary drug resistance exists prior to any given treatment, acquired resistance occurs after initial therapy. Unfortunately, the majority of patients will likely develop resistance at a certain point of treatment. For example, 50–70% of patients with adenocarcinoma relapse following surgery with a chemoresistant phenotype (Castells et al., 2012), and approximately 20% of adults with acute lymphoblastic leukemia suffer from primary resistance to treatment (Testi et al., 1992; Giona et al., 1994; Thomas et al., 1999; O’Connor et al., 2011). In addition, primary resistance has been recognized in nearly 50% of all cancer patients in the 1990s (Pinedo and Giaccone, 1998). Therefore, the design of anti-cancer drugs that are fully effective necessitates a better understanding of the mechanisms by which cancer cells elude treatment. Here we will discuss several features of drug resistant cells including modification of drug transport, mutation of extracellular receptors, amplification and mutation of drug targets as well as related topics. Additionally, we will briefly address the important question of how resistant cell populations emerge.

## MECHANISMS OF DRUG RESISTANCE

**FIGURE 1:**
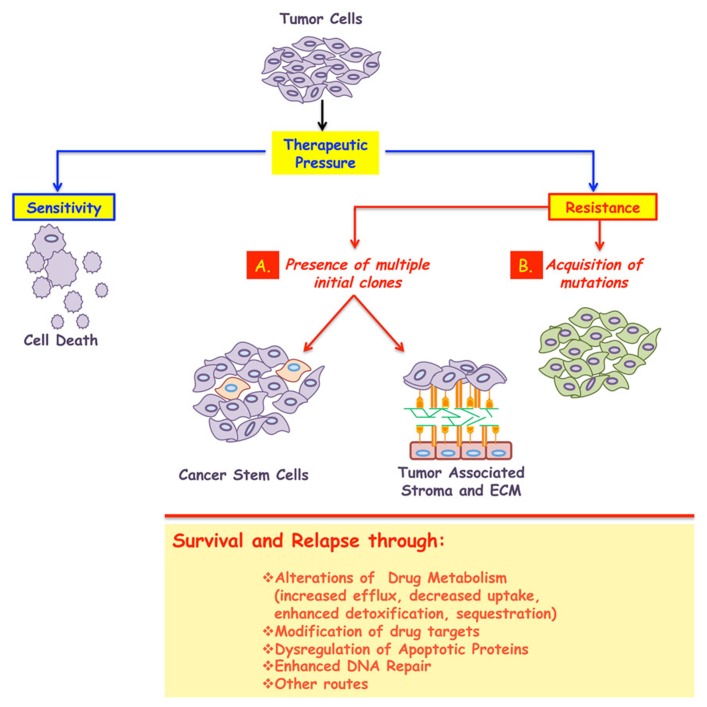
**A major impediment in the treatment of cancer is the development of resistance.** While most tumors initially respond to the given therapy, the majority will relapse following treatment, and in some cases resistance even predates clinical intervention. Therefore cancer resistance can be classified in to two broad classes: primary or acquired. In both cases, the emergence of resistant cells could be due to, at least, two mechanisms: **(A)** presence of multiple initial clones some of which emerge as dominant after treatment. These subpopulations could possess stem-like characteristics and/or use their interactions with the surrounding microenvironment to enter into a dormant state, thus surviving the insult of therapy. **(B)** Acquisition of stochastic alterations within the cancer cells *per se*. In all cases, the surviving population is less likely to respond to any further therapy and will be responsible for the minimal residual disease and cancer relapse. The biochemical underpinnings of resistance include: alterations to drug metabolism, increased drug efflux, decreased drug uptake, modification of the drug targets, amplification of targeted protein, genetic rewiring, enhanced DNA repair, inactivation of apoptotic proteins, or activation of anti-apoptotic ones, among others.

Both primary and acquired resistance can be caused by alterations to drug metabolism (sequestrations or enhanced detoxification) or modifications to the drug targets (Gottesman, 2002; Gatti and Zunino, 2005; Teicher, 2006; Wilson et al., 2006; Ullah, 2008). A brief overview of these mechanisms supported with examples of clinical relevance are presented below (**Figure 1**).

Perhaps the most studied mode of resistance involves drug metabolism, including its uptake, efflux, and detoxification. The means by which drugs enter cells depend on their chemical nature, and it mainly necessitates the use of receptors, which they bind to and transmit their effects without cellular entry, or transporters, which allow their cellular entry (Gottesman, 2002). At this level, resistance can result from mutations that modify activity or reduce the expression of surface receptors and transporters. For instance, mutations or reduced expression of the extracellular receptor smoothened (Yauch et al., 2009; Atwood et al., 2012; Kasper and Toftgard, 2013), nucleoside transporters (Galmarini et al., 2001; Damaraju et al., 2003) or one or both folate transporters (Longo-Sorbello and Bertino, 2001) result in defective uptake of cyclopamine, nucleoside drugs, such as cytarabine, and toxic folate analogs, such as methotrexate, respectively. On the other hand, enhanced drug efflux is frequently caused by increased expression of ATP binding cassette (ABC) membrane transporters (Gottesman et al., 2002). Among the 48 known ABC transporters in humans, elevation of three members, P-gp (*MDR1* gene product), Multidrug resistance-associated protein 1 (MRP1) and mitoxantrone resistance protein [MXR; also known as breast cancer resistance protein (BCRP) or placenta ABC protein (ABC-P)], have been correlated with cancer chemoresistance to various drugs (Gottesman, 2002; Gottesman et al., 2002). For instance, P-gp transports a wide variety of hydrophobic anti-cancer drugs such as vinblastine, doxorubicin, vincristine, and taxol, and therefore its increased expression has been correlated with resistance to these (Gottesman et al., 2002). MRP1 on the other hand, transports negatively charged natural-product drugs in addition to drugs that have been modified by the conjugation of glutathione (GSH), glucuronic acid or sulfate (Jedlitschky et al., 1996; Hipfner et al., 1999; Konig et al., 1999; Borst et al., 2000); while, MXR overexpression has been correlated with resistance to topoisomerase I inhibitors, anthracyclines, and mitoxantrone (Gottesman, 2002). As can be seen, these factors comprise a major site for the development of drug resistance.

To exert their cytotoxic effects, many anti-cancer drugs must undergo metabolic activation. For instance, cytarabine (also known as AraC), a nucleoside drug widely used for the treatment of acute myelogenous leukemia (Sampath et al., 2006), necessitates initial phosphorylation by deoxycytidine kinase to cytarabine-monophosphate which is subsequently phosphorylated to the active form cytarabine triphosphate. To circumvent the effects of these drugs, cancer cells develop resistance through decreased drug activation (Kufe and Spriggs, 1985; Bardenheuer et al., 2005). This occurs via the downregulation or mutation of enzymes involved in this metabolic pathway, such as deoxycytidine kinase in the case of cytarabine (Sampath et al., 2006). Drug inactivation can also play a major role in the development of resistance. These mechanisms include, for example, conjugation of the drug to GSH, a powerful anti-oxidant that protects the cells against the damaging effects of reactive oxygen species (Wilson et al., 2006). GSH conjugation to platinum drugs, such as oxaliplatin and cisplatin used in the treatment of various types of cancers, renders them substrates for ABC transporters which enhances drug efflux (Meijer et al., 1992; Ishikawa and Ali-Osman, 1993). Furthermore, the topoisomerase I inhibitor, irinotecan, used for treating colon cancer, have been shown to become inactivated via phase I drug metabolizing enzymes, CYP450 (Xu and Villalona-Calero, 2002). Finally, binding of platinum drugs, particularly cisplatin, to metallothionein (MT), a small cysteine-rich protein, is another means of drug inactivation (Kelley et al., 1988; Kasahara et al., 1991).

Many cancer cells develop an overreliance or dependency on an oncogene. This is referred to as oncogene addiction (Arber et al., 1997; Weinstein, 2002; Weinstein and Joe, 2006; Sharma and Settleman, 2007). Targeting such oncogenes, provided a basis for the development of targeted therapies. Examples of such targeted therapies include: (i) imatinib targeting BCR/ABL tyrosine kinase in CML (Hughes et al., 2003), (ii) gefitinib and erlotinib targeting the epidermal growth factor receptor (EGFR) tyrosine kinase domain in non-small cell lung carcinoma (Lynch et al., 2004; Shepherd et al., 2005; Taron et al., 2005), and (iii) trastuzumab targeting human epidermal growth factor receptor-2 (HER-2) receptor in breast carcinomas (Slamon et al., 2001; Piccart-Gebhart et al., 2005). Unfortunately, the long term effectiveness of these drugs is hindered by the development of drug resistance due to mutation of the targeted protein (Gioeli, 2011; Wong and Lee, 2012). In the case of BCR/ABL and EGFR inhibitors, resistance emerges as a result of mutations occurring at the gatekeeper residues of the kinase domain which disables drug binding (Gorre et al., 2001; Blencke et al., 2003; Kobayashi et al., 2005; Pao et al., 2005; Soverini et al., 2005; Balak et al., 2006; Jabbour et al., 2006, 2008; Nicolini et al., 2006; Apperley, 2007; Costa et al., 2007; Bean et al., 2008; Gioeli, 2011). Furthermore, it has been demonstrated that resistance mutations can be detected prior to treatment in small subpopulations of tumor cells suggesting that these mutant forms were selected via the targeted therapy used (Hofmann et al., 2003; Toyooka et al., 2005; Inukai et al., 2006). In essence, understanding how mutations in the target proteins confer resistance enables the development of new therapeutic approaches to surmount resistance. For instance, second generation CML inhibitors have been developed based on mutational studies of patients who have become Gleevec resistant.

Other mechanisms by which cancerous cells circumvent the effects of targeted inhibitors have also been described, including amplification of alternative oncogenes or inactivation of alternative survival pathways (le Coutre et al., 2000; Engelman et al., 2007). In some cases, targeting of one protein alone (that cells are showing dependency on) can become ineffective because another parallel pathway supports tumor survival. In this case, the two pathways develop a synthetic lethal relationship (Hartman et al., 2001; Tucker and Fields, 2003). This way, the loss/inactivation of one of these genes would be supported by the other pathway and for the most effective treatment, one would need to target both pathways (Luo et al., 2009; Nijman, 2011).

An example of new pathways emerging once another pathway is targeted comes from the work of Isoyama et al. (2012), showed that acquired resistance to phosphatidylinositol 3-kinase (PI3K) inhibitors (such as ZSTK474) was due to the upregulation of insulin-like growth factor 1 receptor (IGF1R) pathway and that inhibition of this pathway with selective IGF1R inhibitors reverses the acquired PI3Ki resistance phenotype (Isoyama et al., 2012). Additionally, resistance could result from evasion of apoptotic pathways triggered by the acquisition of either inactivating mutations in genes coding for apoptotic proteins, such as p53, or activating mutations in genes coding for anti-apoptotic proteins, such as B cell lymphoma 2 (Bcl-2; Teicher, 2006). Indeed p53 mutations have been correlated with *de novo* resistance to doxorubicin treatment in patients with advanced breast cancer, as well as resistance to anthracyclines in a mouse sarcoma tumor model (Aas et al., 1996; Levine, 1997).

Another excellent example of this phenomenon (i.e., synthetic lethality) is seen in breast and ovarian cancers carrying mutations in the *BRCA1* and *BRCA2* genes, important mediators of DNA double-strand break (DSB) repair. When the poly (ADP-ribose) polymerase (PARP) protein, which is involved in different cellular processes including DNA repair, was targeted in these tumors, selective cancer cell toxicity was achieved (Bryant et al., 2005; Farmer et al., 2005). Several PARP inhibitors (PARPi) are currently being tested in clinical trials, such as iniparib (phase III ongoing; Guha, 2011) and veliparib cancer (Trudeau et al., 2006; Palma et al., 2008, 2009; Kummar et al., 2011), among others. However, despite the promising results these inhibitors showed, whether used as a mono- or combinatorial therapy (Juvekar et al., 2012; Kummar et al., 2012; Riffell et al., 2012), cancer cells once again were capable of evolving resistance to PARPi in preclinical and clinical settings (Chiarugi, 2012; Montoni et al., 2013). The mechanisms of resistance to these inhibitors have been grouped in to at least four categories, as summarized recently (Montoni et al., 2013). But perhaps the most distinct of these, was the ability of cancer cells to revert sensitivity to PARPi by acquiring deletion of the mutation in BRCA gene, thus restoring its function and the subsequent repair of DSBs.

## DEVELOPMENT OF CROSS RESISTANCE

An important feature of drug resistance, is that development of resistance to one drug can lead to resistance to other drugs (Ullah, 2008). For instance, loss of a drug transporter can lead to resistance to structurally diverse compounds that utilize it or elevation of ABC transporters resulting from one therapy will influence the efficacy of many other compounds. Since this multidrug resistance phenotype correlates with poor chemotherapy response, drug development strategies to overcome this problem are being designed. These include drugs that are not recognized by transporters and therefore evade efflux, efflux inhibitors, drugs that are selectively lethal to P-gp expressing cells, etc. (Hall et al., 2009; Kelly et al., 2011; Nobili et al., 2012). But, perhaps resistance is not useless after all, as Hall et al. (2009) proposed. The alternative strategy to treat the progeny of the drug imposed Darwinian selection process is to identify their new “Achilles’ heel,” where resistance to the first given drug conferred a hypersensitivity to an alternate cytotoxic agent to which parental cells were not sensitive to. A phenomenon referred to as “collateral sensitivity”, which could be considered as a type of synthetic lethality as well since the same genetic alteration that rendered the cells resistant to one drug now sensitizes them to another (Hall et al., 2009; Pluchino et al., 2012).

## WHERE DO RESISTANT CELLS COME FROM?

The development of human cancers is a complex multistage process involving accumulation of both genetic and epigenetic alterations over time (Caulin and Maley, 2011). As a consequence, a single tumor is comprised of heterogeneous populations of cells with distinct genetic fingerprints (Heppner et al., 1978; Marusyk and Polyak, 2010; Michor and Polyak, 2010). As the tumor progresses, some cells undergo genetic alterations, with selection of those having a superior growth advantage in a given context. An excellent example of tumor heterogeneity is provided by breast cancer studies (Schvimer et al., 1995; Shen et al., 2000; Wild et al., 2000). Wild et al. (2000) demonstrated that about 97% of epithelial breast carcinomas possess high levels of intra-tumor diverseness. The relevance of this innate heterogeneity is seen in cancer resistance. Since cancer cell selection obeys the Darwinian law of evolution, hence, under therapeutic pressure, those populations that are most adaptive or resistant to treatment will be selected for. These clones will then dominate and populate the tumor rendering it highly resistant to the given therapy (Williams and Nesse, 1991; Nesse, 2001; Breivik, 2005; Crespi and Summers, 2005; Lichtenstein, 2005; Monceviciute-Eringiene, 2005; Greaves, 2007). The selection process can be rationalized by, at least, two mechanisms. First, the emergence of a dominant cellular population after drug selection since it possesses some favorable characteristics such as a mutated drug binding site (Zhang et al., 2006; O’Brien et al., 2007; Ricci-Vitiani et al., 2007). The second mechanism involves the acquisition of stochastic alterations within the cancer cells which provide a survival advantage (Campbell et al., 2008; Stratton et al., 2009; Negrini et al., 2010; The International Cancer Genome Consortium, 2010; Shen, 2011). The advantage itself, e.g., a mutation in a drug binding site or alteration in drug transporters (as just two examples) could be the same for either of these mechanisms. What is different is the underlying process to generate these biochemical differences.

Two known models, the cancer stem cell (CSC) model, and the environment-mediated drug resistance (EMDR) model, which are not mutually exclusive, could explain the origin of resistant cells. In the CSC model, rare populations of cancer stem cells possess tumor-initiating properties (Teicher, 2006; Nguyen et al., 2012). It is thought that CSCs diverge from normal tissue stem cells or from more-differentiated progenitor cells through dysregulation of self-renewal pathways. Beside modulation of molecular mechanisms, such as increased efficiency of DNA repair (Potten et al., 2002; Cai et al., 2004; Park and Gerson, 2005), changes in cell cycle parameters (Venezia et al., 2004), overexpression of anti-apoptotic proteins (Wang et al., 2003) or drug transporters (Gottesman et al., 2002; Krishnamurthy et al., 2004), etc., resistance of CSCs could be due to their quiescent nature (Teicher, 2006). Thus, in this case, the cell population is present and is difficult to target using traditional chemotherapy strategies many of which depend on active cell cycling.

In the EMDR model, resistance emerges as the cancer cells use their interactions with the surrounding microenvironment to enter into a quiescent or dormant state as a means of circumventing the effects of the given therapy. Under the drug imposed selection pressure, these cells remain in their protective shelter, undergoing genetic changes until they ultimately reach a more permanent acquired resistance phenotype and in turn, alter their surrounding microenvironment (Braun et al., 2000; Meads et al., 2009). These surviving populations, which may or may not be CSCs, can contribute to minimal residual disease (MRD) and cancer relapse (Matsunaga et al., 2003; Bidard et al., 2008; Meads et al., 2009). The EMDR model is relevant to both hematopoietic and metastatic epithelial malignancies. EMDR could be mediated by either soluble or cell adhesion-related microenvironmental factors. Soluble factor-mediated drug resistance occurs through induction of gene transcription within the tumor cells by cytokines, chemokines, or growth factors secreted by neighboring stroma-like fibroblasts (Meads et al., 2009). One of the known mediators of this resistance mechanism is interleukin-6 (IL-6), whose increased secretion has been correlated with resistance to various cytotoxins both in *in vitro* and *in vivo *models. This includes, for instance, resistance to bortezomib in multiple myeloma and to etoposide and cisplatin in hormone-independent prostate carcinomas (Borsellino et al., 1995, 1999; Frassanito et al., 2001; Voorhees et al., 2007). Further, cell adhesion-mediated drug resistance is triggered by the adhesion of integrins from tumor cells to stromal fibroblasts or to components of the surrounding extracellular matrix. Molecularly, this process could be due to many scenarios including (i) degradation of apoptotic proteins or (ii) enhanced stability or altered subcellular distribution of anti-apoptotic proteins and cell cycle regulators (Hazlehurst et al., 2001, 2007; Shain et al., 2002, 2009; Lwin et al., 2007). One example is provided by studies into melphalan resistance. In this case, the cancerous cells tend to use their adhesion to fibronectin in the surrounding microenvironment to reduce the endogenous levels of the proapoptotic BH3-only Bcl-2 family member, Bim1, thus conferring resistance by disabling apoptosis (Hazlehurst et al., 2003; Hanahan and Weinberg, 2011). From a clinical point of view, it is thought that combining current therapies with inhibitors of EMDR pathways could enhance the effectiveness of the treatment (Croix et al., 1996; Weaver et al., 1997; Hazlehurst et al., 2000; White et al., 2004; Lwin et al., 2007). A proof-of-principle example was demonstrated by the combination of melphalan, a DNA alkylating agent used in the treatment of multiple myeloma and ovarian carcinomas, with an anti-integrin α-4 antibody (natalizumab) which significantly inhibited myeloma growth and reduced tumor burden in patients (Mori et al., 2004; Engelhardt and Kappos, 2008).

## CONCLUSION

Resistance to drugs continues to be a major problem in oncology affecting the majority of cancer patients. Here we provide many examples of how cells become resistant to various drugs including alteration in drug metabolism, modification of drug targets, and genetic rewiring of cells to bypass targeted pathways. A better understanding of oncogene networks and oncogene cooperativity will likely improve therapeutic strategies by identifying optimal combinations based on the genetic lesions in the tumors. Importantly, tumors are highly heterogenous and this heterogeneity may well substantially contribute to primary or acquired resistance. Armed with a greater understanding of the mechanisms of drug resistance will undoubtedly lead to more long term remissions and hopefully cures.

## Conflict of Interest Statement

The authors declare that the research was conducted in the absence of any commercial or financial relationships that could be construed as a potential conflict of interest.
